# Death Following Lumbar Puncture

**Published:** 1915-11

**Authors:** 


**Death Following Lumbar Puncture.** Aides-Majors, Blondin and Senechal, Bull, et Mem. de la Soc. de Med. de Paris, May 14. The case was that of a soldier wounded by a bomb with loss of substance of the cranium, 3.5 c.m. in diameter, external and superior to the right orbital region. There was some loss of cerebral substance and clots but no mention of loss of function. On account of headache, lumbar puncture was practiced 12 days after injury, 15 c.c. of very slightly lemon colored liquid issuing in a jet. Shortly afterward, he died of puomonary oedema although he had not complained of any symptoms and had objected to rest in bed. Necropsy confirmed the observations, showed healing without suppuration but with formation of clots and did not secure the missile, though there was no wound of exit. Levy-Valensi, L'Hop. May 1914, has reported death following puncture, about 30 cases being recorded. The authors, however, do not consider that the puncture could have been the essential cause of death.

